# Prevalence, knowledge and perception of self-medication practice among undergraduate healthcare students

**DOI:** 10.1186/s40545-021-00331-w

**Published:** 2021-06-10

**Authors:** Wuraola Akande-Sholabi, Amen. T. Ajamu, Rasaq Adisa

**Affiliations:** Department of Clinical Pharmacy and Pharmacy Administration, Faculty of Pharmacy, University of Ibadan, Ibadan, Nigeria

**Keywords:** Self-medication practice, Antimicrobial resistance, Healthcare students, University undergraduate

## Abstract

**Background:**

Globally, self-medication is a common practice, and an increasingly perceived necessity to relieve burdens on health services. However, inappropriate self-medication may result to reduced health outcomes, increased antimicrobial resistance and economic waste. Healthcare students are the future health professionals who will be consistently responsible for educating the public on rational use of medication. This study therefore aimed to assess the prevalence, knowledge and perception of self-medication practices among healthcare students.

**Methods:**

A cross-sectional study was carried out among 866 healthcare students in a Nigerian University, comprising medical, nursing and pharmacy students. Information was garnered from respondents using a self-administered questionnaire. Data were summarized with descriptive statistics, while Chi-square and logistic regression tests were used for categorical variables at *p* < 0.05.

**Results:**

Mean age was 21 ± 2.8 years, and female respondents were 447 (51.6%). Prevalence of self-medication among respondents was 473 (54.6%). A total of 288 (55.3%) demonstrated good knowledge of self-medication practices, comprising 250 (52.2%) among those who have previously self-medicated and 229 (47.8%) among those who had not. Reasons for engaging in self-medication practices were mentioned to include treatment of minor ailments (357; 32.4%), while 248 (22.5%) believed they had the medical knowledge of what to use. Analgesic (353; 30.1%), antimalarial (352; 30.0%), and antibiotics (182; 15.5%) were the commonest classes of medication used for self-medication. Headache (363; 18.4%), malaria (334; 16.9%), and cough (184; 9.3%) were the most frequently treated conditions. More than half (281; 59.4%) of the respondents’ purchased their self-medicated drugs from the community pharmacy. Gender and respondents’ disciplines were found to be the independent predictors for good knowledge of self-medication practice.

**Conclusion:**

Prevalence of self-medication among the studied healthcare students is moderately high, while approximately half demonstrates good knowledge and perception of self-medication practices. Stimulation for self-medication practice largely arise from the perception of treating minor ailments. This underscores a need for advocacy on responsible self-medication practice during the formal training of these future health professionals, in order to avert its imminent/widespread negative consequences.

## Introduction

Globally, self-medication is increasingly being considered as a component of self-care [1]. World Health Organization (WHO) defines self-care as what people do by themselves to keep their health, prevent and treat illness [2], while International Pharmaceutical Federation [3], supported by World Health Organization [4] defines self-medication (SM) as the self-administration of a medication in the absence of a current prescription and/or without consulting a healthcare professional. Self-medication (SM) with either over-the-counter medications or prescribed medications including antibiotics is a common practice in many developing countries, and partly in some developed countries [5]. The reason(s) for engaging in self-medication practice in developing countries have been reported to include lack of medical insurance, expensive hospitals visits/consultation fees, easy public access to the prescribed medications [5], suggestions of friends, inexpensiveness of the practice and previous experience [6–9]. Generally, SM is regularly used for minor ailments such as headache, fever, sore throat, gastrointestinal tract problems, respiratory problems, skin disorders, ear symptoms among others [10–15], and WHO recognizes SM as a viable tool for achieving universal health coverage [16, 17].

Self-medication could offer several advantages to patients including quick access to treatment, self-independence in alleviating symptoms, reduction in the cost of accessing healthcare and frequency of visits to health centers; and also, to the community, its advantages include saving medical resources, decreasing absence from work, declining pressure on medical services and providing more time for critical conditions [18]. Notwithstanding its various advantages, self-medication, especially if unguided could results into possible risks at the individual level such as incorrect diagnosis, serious adverse effects, increased antimicrobial resistance, dangerous food and drug interactions, as well as drug misuse and abuse. Also, at the community level, unguided self-medication may lead to increased drug induced disease and public expenses [18, 19]. The negative consequences of SM can be largely felt in many developing countries with limited resources, low literacy level and healthcare amenities, as well as the huge populace who neither have access to information nor satisfactory knowledge regarding therapy, dosage and duration of use or side effect [18–20]. In Nigeria for instance, the sales of both over-the-counter (OTC) and prescription-only medicine by roadside hawkers, and various unregistered and registered proprietary and patent medicine vendors is common [21], largely because of weak enforcement of drug regulations [22]. The unregulated sales of these products, may incessantly trigger self-medication practice among the general populace. However, a responsible/guided self-medication may still be envisaged, whereby the patient treat his illness or symptom with medicine which are approved and available without prescription, but which are safe and effective when used as directed [4].

Previous studies have documented that, influencing the prescribing conduct as well as knowledge of the healthcare professionals can encourage responsible self-medication [6, 7, 13, 14]. Thus, creating awareness frequently and providing extensive formal training on the concept of responsible self-medication for healthcare students who are the future health professionals may potentially be an efficient approach to circumvent the unwarranted consequences of self-medication. However, to partly achieve this, there may be a need to probe into the knowledge and extent of involvement of healthcare students in self-medication practices.

Most of the previous studies on self-medication in Nigeria focused on general populations [8–10, 21, 23–25], with a paucity of information on healthcare students. Thus, this study aims to assess the prevalence, knowledge and perception of self-medication practices among healthcare students, while the possible reasons for engaging in such practice were also explored.

## Methods

### Study design and setting

This study was a university-based cross-sectional study using a self-administered questionnaire. It was conducted among undergraduate students at the University of Ibadan, in the Faculty of Pharmacy, Departments of Medicine and Surgery, as well as Nursing, between August and November 2019. Presently in Nigeria, the Bachelor of Pharmacy and Bachelor of Nursing degrees are a 5-year program, while the Bachelor of Medicine and Surgery degree is a 6-year program.

### Data collection instrument

The questionnaire used for this study was designed by the researchers after a thorough review of similar studies [10, 26–28], as well as utilizing researchers’ proficiency. The questionnaire comprised three sections. Section A evaluated socio-demographic characteristics of the students such as age, gender, level of study and course of study. Section B consisted of questions on students’ level of knowledge and perception on self-medication, while Section C consisted of questions on the participants’ practice of self-medication. The questions were evaluated on a 5-point Likert rating scale from strongly agree (5) to strongly disagree (1) to explore and evaluate students’ knowledge and perception of self-medication practice. The overall score for the knowledge and perception questions were categorized into ‘good’ and ‘poor’ depending on respondents’ score in each domain. For the 8-item statements on knowledge with 5-point Likert scale response, a total score of at least 32 (≥ 80%) out of the maximum obtainable score of 40 was categorized as ‘good’ knowledge, while a knowledge score < 32 (< 80%) was categorized as ‘poor’ knowledge. For the 3-item statements on perception with 5-point Likert scale response, a total score of at least 12 (≥ 80%) out of the maximum obtainable score of 15 was categorized as ‘good’ perception, while a score < 12 was categorized as ‘poor’ perception. The binary categorization of knowledge and perception scores was adapted from Bloom’s cut-off criteria and other similar studies [29–31].

### Inclusion and exclusion criteria

Eligible participants were registered undergraduate students of Medicine and Surgery, Pharmacy and Nursing, for the 2018/2019 academic session. Participants must have also given voluntary informed consent to partake in the study. Students who were absent in the classes during the period of questionnaire administration, as well as the non-consenting individuals were excluded.

### Sample size determination

Based on the population of 1521 registered students for the 2018/2019 academic session, comprising Pharmacy (360), Medicine and Surgery (881), and Nursing (280) at 95% confidence level and 5% alpha error, a sample size of 316 was obtained using Yamane’s formula [32]. Additionally, to cater for the possibility of a low response rate, which is not uncommon among students, an attrition rate close to 200% was allowed.

### Sampling and data collection procedure

Students were approached after a major lecture identified across the levels in each faculty/departments, purpose and objectives of the study were explained to the students immediately at the end of the identified major classes. Each questionnaire, which took about 12–15 min to be completed was subsequently administered only to the consented individuals. This was done in the period allocated for questionnaire administration for each Faculty or Department. The questionnaires were retrieved after completion and inspected for completeness and correctness. Study participation was voluntary and the students were informed of the possibility of withdrawing from the study anytime. Response anonymity and confidentiality were reiterated to the respondents. Measures were put in place to prevent multiple filling of the questionnaire by the respondents. This was achieved by coding each questionnaire administered to the students from each department to avoid duplication.

### Pretest and content validation

Content validity was established by three scholars with expertise in the subject area, to ascertain the inclusiveness of question-items in line with the objectives of the study. Thereafter, pretest was carried out by administering the questionnaire to thirty students randomly selected from the Pharmacy, Nursing, and Medical and Surgery departments. These students were exempted from the main study. Feedback from these students on clarity of the questions or statements in the questionnaire led to some modifications in the questionnaire, such as significant reduction in the number of open-ended questions.

### Statistical analysis

Data were coded, cleaned, and analyzed using the IBM Statistical Package for Social Sciences (version 23). Descriptive statistics such as frequency, percentages, mean, and standard deviation were used to summarize the data. The internal consistency/reliability of the 11-items self-medication knowledge and perception questions was determined using Cronbach alpha test, with a value of 0.68. Association between demographic variables and respondents’ engagement in self-medication practice or not was evaluated with Chi-square, while binary logistic regression was used to ascertain the predictors of good or poor knowledge and perception on self-medication practice. The level of significance was set at *p* < 0.05.

## Results

### Demographic characteristics of respondents

Of the 960 copies of questionnaires distributed among the respondents, 866 were completed and included in the analysis given a response rate of 90.2%. This comprised, Medicine and Surgery (436; 50.3%), Pharmacy (275; 31.8%) and Nursing (155; 17.9%) students. Mean age was 21 ± 2.8 years, and 447 (51.6%) were females. The prevalence of self-medication practice was highest among females (261; 58.4%) and students aged > 20 years (259; 56.2%). Also, self-medication practice among the healthcare students was in the order of Medicine and Surgery (259; 59.4%) > Nursing (78; 50.3%) > Pharmacy (136; 49.5%), *p* = 0.017. Year 5 and Year 6 students constituted those who had largely engaged in self-medication, 116 (63.0%) and 44 (81.5%), respectively (*p* = 0.000). In all, the prevalence of self-medication practice among the respondents was 473 (54.6%) (Table 1).

**Table 1:** Distribution of respondents demographic characteristics based on the practice of self-medication (n = 866)

Variable	Engagement in self-medication, YES *n* (%) *n* = 473 (54.6)	No self-medication *n* (%) *n* = 393 (45.4)	Chi-square	*p*-value
Gender				
Male	212 (50.6)	207 (49.4)	5.298	0.021*
Female	261 (58.4)	186 (41.6)		
Age (year)				
≤ 20	214 (52.8)	191 (47.2)	4.193	0.123
> 20	259 (56.2)	201 (43.8)		
Department				
Nursing	78 (50.3)	77 (49.7)	8.14	0.017*
Pharmacy	136 (49.5)	139 (50.5)		
Medicine and surgery	259 (59.4)	177 (40.6)		
Year of study				
Year 1	62 (49.6)	63 (50.4)	33.631	0.000*
Year 2	66 (51.2)	63 (48.8)		
Year 3	105 (56.1)	82 (43.9)		
Year 4	80 (42.8)	107 (57.2)		
Year 5	116 (63.0)	68 (37.0)		
Year 6	44 (81.5)	10 (18.5)		

*Statistically significant (*p* < 0.05)

### Knowledge and perception about self-medication practice among respondents

Table 2 shows the assessment of respondents’ knowledge and perception on the practice of self-medication. A total of 479 (55.3%) had score ≥ 80% indicating ‘good’ knowledge; comprising 250 (52.2%) among those who had self-medicated and 229 (47.8%) among those who had not. Over half (223; 57.6%) of the respondents who had self-medicated constituted those with ‘poor’ knowledge compared to those who had not (164; 42.4%). Moreover, a total of 584 (67.4%) had score ≥ 80% indicating ‘good’ perception. There was no statistically significant difference among respondents who had either self-mediated or not in respect of knowledge (*χ*^2^ = 2.547; *p* = 0.111) and perception (*χ*^2^ = 0.020; *p* = 0.887).

**Table 2:** Knowledge and perception about self-medication practice among respondents (*n* = 866)

				SA		A		U		D		SD
Knowledge question^a^												
1		Self-medication is defined as “*the taking of drugs, herbs or home remedies on one's own initiative, or on the advice of another person, without consulting a doctor or pharmacist.”*		557 (64.3%)		277 (32.0%)		7 (0.8%)		10 (1.2%)		15 (1.7%)
2		Using an old prescription to treat a recurring condition is self-medication		318 (36.7%)		411 (47.5%)		71 (8.2%)		50 (5.8%)		16 (1.8%)
3		Using left-over medications from a prior properly diagnosed illness to treat a recurrent one is self-medication		303 (35.0%)		403 (46.5%)		79 (9.1%)		65 (7.5%)		16 (1.8%)
4		A physician’s prescription is necessary before the purchase of any medication		329 (38.0%)		294 (33.9)		70 (8.1)		132 (15.2)		41 (4.7%)
5		It is possible to correctly treat illnesses without the physician’s prescription		59 (6.8%)		415 (47.9%)		159 (18.4%)		151 (17.4%)		82 (9.5%)
6		Using prescription only medications without the doctor’s prescription can cause complications		310 (35.8%)		417 (48.2%)		71 (8.2%)		48 (5.5%)		20 (2.3%)
7		Self-medication can lead to drug dependence and adverse drug reaction		343 (39.6%)		403 (46.5%)		76 (8.8%)		29 (3.3%)		15 (1.7%)
8		Self-medication can cause pathogen resistance		356 (41.1%)		374 (43.2%)		80 (9.2%)		36 (4.2%)		20 (2.3%)
Cut-off score		Remark		Self-medication		No Self-medication						
< 80		Poor knowledge		223 (57.6%)		164 (42.4%)						
≥ 80		Good knowledge		250 (52.2%)		229 (47.8%)						
				*χ*^2^ = 2.547 *p*-value = 0.111								
Perception question^b^												
1	The use of medications without the physician’s prescription is safe		22 (2.5%)		46 (5.3%)		121 (14.0%)		401 (46.3%)		276 (31.9%)	
2	The use of left-over medication from friends and family is safe		20 (2.3%)		28 (3.2%)		65 (7.5%)		373 (43.1)		380 (43.9%)	
3	Self-medication cannot cause serious health hazards		48 (5.5%)		88 (10.2%)		83 (9.6%)		315 (36.4%)		332 (38.3%)	
Cut-off score	Remark		Self-medication		No Self-medication							
< 80	Poor perception		155 (55.0)		127 (45.0)							
≥ 80	Good perception		318 (54.5)		266 (45.5)							
			*χ*^2^ = 0.020 *p*-value = 0.887									

^a^Maximum obtainable score = 40; %individual score = score obtained by an individual ÷ by total obtainable score × 100. Strongly agree (SA) = 5, Agree (A) = 4, Undecided (U) = 3, Disagree (D) = 2, Strongly disagree (SD) = 1

^b^Maximum obtainable score = 15; %individual score = score obtained by an individual ÷ by total obtainable score × 100. Strongly agree (SA) = 5, Agree (A) = 4, Undecided (U) = 3, Disagree (D) = 2, Strongly disagree (SD) = 1

### Determinants of knowledge and perception on self-medication practice among the respondents

Gender and respondents’ disciplines were found to be independent predictors of good knowledge. Females were 1.4 times more knowledgeable about self-medication (AOR: 1.43 [CI: 1.059–1.953], *p* = 0.02) than males. However, students of Pharmacy had lower odds for knowledge (AOR: 0.621 [CI: 0.401–0.961], *p* = 0.03), and perception (AOR: 0.577 [CI: 0.365–0.913], *p* = 0.019) on self-medication practice than the students of Nursing and Medicine and Surgery. Details are in Table 3.

**Table 3:** Determinants of knowledge and perception of self-medication practice among respondents (n = 866)

Variables	Knowledge on self-medication		Unadjusted odds ratio		Adjusted odds ratio	
	Good (%)	Poor (%)	OR (95% CI)	*p*-value	OR (95% CI)	*p*-value
Gender						
Male	214 (51.1)	205 (48.9)	1	0.015*		0.020*
Female	265 (59.3)	182 (40.7)	1.395 (1.066–1.825)		1.431 (1.059–1.935)	
Age-group (years)						
≤ 20 ref	223 (55.1)	182 (44.9)	1	0.890		0.642
> 20	256 (55.5)	205 (44.5)	1.019 (0.779–1.333)		0.909 (0.607–1.361)	
Department						
Nursing ref	97 (62.6)	58 (37.4)	1			
Pharmacy	135 (49.1)	140 (50.9)	0.577 (0.386–0.862)	0.007*	0.621 (0.401–0.961)	0.033*
Medicine and surgery	247 (56.7)	189 (43.3)	0.781 (0.536–1.139)	0.199	1.046 (0.685–1.598)	0.834
Year of study						
Year 1	69 (55.2)	56 (44.8)	1		1	
Year 2	72 (55.8)	57 (44.2)	1.025 (0.625–1.682)	0.922	0.989 (0.593–1.650)	0.966
Year 3	93 (49.7)	94 (50.3)	0.803 (0.510–1.265)	0.344	0.781 (0.488–1.251)	0.304
Year 4	117 (62.6)	70 (37.4)	1.357 (0.856–2.150)	0.194	1.624 (0.950–2.775)	0.076
Year 5	103 (56.0)	81 (44.0)	1.032 (0.653–1.630)	0.892	1.244 (0.692–2.236)	0.466
Year 6	25 (46.3)	29 (53.7)	0.274 (0.700–1.328)	0.274	0.691 (0.321–1.485)	0.344

	Perception on self-medication		Unadjusted odds ratio		Adjusted odds ratio	
	Good (%)	Poor (%)	OR (95% CI)	*p*-value	OR (95% CI)	*p*-value
Gender						
Male	282 (67.3)	137 (32.7)	1	0.935		0.780
Female	302 (67.6)	145 (32.4)	1.012 (0.761–1.345)		0.956 (0.697–1.312)	
Age-group (years)						
≤ 20 ref	278 (68.6)	127 (31.4)	1	0.478		0.232
> 20	306 (66.4)	155 (33.6)	0.902 (0.678–1.200)		0.772 (0.506–1.179)	
Department						
Nursing ref	111(71.6)	44 (28.4)	1			
Pharmacy	165 (60.0)	110 (40.0)	0.595 (0.389–0.909)	0.016*	0.577 (0.365–0.913)	0.019*
Medicine and surgery	308 (70.6)	128 (29.4)	0.954 (0.636–1.431)	0.819	0.958(0.611–1.500)	0.850
Year of study						
Year 1	88 (70.4)	37 (29.6)	1		1	
Year 2	88 (68.2)	41(31.8)	0.902 (0.529–1.539)	0.706	0.829 (0.480–1.433)	0.502
Year 3	120 (64.2)	67 (35.8)	0.753 (0.463–1.225)	0.253	0.740 (0.449–1.220)	0.238
Year 4	131(70.1)	56 (29.9)	0.984 (0.599–1.614)	0.948	1.226 (0.694–2.165)	0.483
Year 5	115 (62.5)	69 (37.5)	0.701 (0.431–1.140)	0.152	0.895 (0.484–1.656)	0.725
Year 6	12 (22.2)	42 (77.8)	1.472 (0.697–3.108)	0.311	1.582 (0.669–3.742)	0.297

*Statistically significant (*p* < 0.05) *CI* confidence interval

### Reasons for engaging in self-medication practice by respondents

The arrays of reasons for engaging in self-medication practice are presented in Table 4. The majority, 357 (32.4%) engaged in the practice for treatment of minor ailments, while 248 (22.5%) believed they had the medical knowledge of what to use and 122 (11.1%) did not want to waste time at the clinic. Other reasons cited included previous experience from old prescriptions (81; 7.3%), inability to afford laboratory investigation costs (66; 6.0%), as well as left-over medications (61; 5.5%) (Table 4).

**Table 4:** Summary of respondents’ reasons for engaging in self-medication practice

^a^Reasons for self-medication (*n* = 1103)	Frequency	Percentage (%)
Treatment of minor ailment	357	32.4
I have medical knowledge of what to use	248	22.5
I do not want to waste time at the clinic	122	11.1
I know what to use from old prescription	81	7.3
I cannot afford laboratory fees	66	6.0
I had left-over medication	61	5.5
The healthcare facility is far from my place of abode	54	4.9
The pain was severe	48	4.4
I cannot wait for laboratory results	37	3.4
I cannot afford consultation fees	29	2.5

^a^Multiple response

### Medications used for self-medication practice and the conditions treated

Analgesic, 353 (30.1%) was the most commonly used class of medication, followed by antimalarial (352; 30.0%), antibiotics (182; 15.5%) and multivitamins, 142 (12.1%). The most frequently treated conditions by self-medication among respondents were headache (363; 18.4%), malaria (334; 16.9%), cough (184; 9.3%), menstrual pain (170; 8.6%) and cold and flu (158; 8.0%). Details are in Table 5.

**Table 5:** Summary of medications used for self-medication and conditions treated by self-medication practice (*n* = 473)

Variable	Frequency	Percentage (%)
^a^Class of medication used for self-medication (*n* = 1172)		
Analgesic	353	30.1
Antimalarial	352	30.0
Antibiotics	182	15.5
Multivitamins	142	12.1
Antihistamine	115	9.8
Slimming pills	19	1.6
Others	9	0.9
^a^Conditions treated by self-medication practice (*n* = 1977)		
Headache	363	18.4
Malaria	334	16.9
Cough	184	9.3
Menstrual pain	170	8.6
Cold and flu	158	8.0
Fever	143	7.2
Sore throat	141	7.1
Diarrhea	113	5.7
Stomachache	105	5.3
Constipation	73	3.7
Skin infections	71	3.6
Vomiting	34	1.7
Infections (toothache, STDs and UTI)	29	1.5
Eye problem	28	1.4
Ear problem	21	1.1
Ulcer	4	0.2
Rhinitis	2	0.1
Toothache	2	0.1
Sinusitis	1	0.05
Nausea	1	0.05

^a^Multiple response

### Source of medications and information on drug dosage regimen for self-medication practice

More than half (281; 59.4%) of the respondents’ purchased their medications from the community pharmacy, followed by proprietary and patent medicine vendor (116; 24.5%), and friends/family (48; 10.1%). Twenty-eight (5.9%) reported to have their medications from the hospital, perhaps representing those who reuse medication from left over of previously prescribed medications. Patient information leaflet (251; 32.3%) was the commonest source of information about dosage and duration of medications, followed by family and friends (172; 22.1%) and classroom/lectures 143 (18.4%) (Table 6).

**Table 6:** Source of information on dosage regimen and medications for self-medication practice

Variable	Frequency	Percentage (%)
Source of medication (*n* = 473)		
Community pharmacy	281	59.4
Patent medicine vendor	116	24.5
Friends/family	48	10.2
Hospital	28	5.9
^a^Source of information on drug dosage regimen (*n* = 777)		
Patient Information leaflet	251	32.3
Family and friends	172	22.1
Classroom	143	18.4
Textbook	132	16.9
Social media	52	6.7
Print media (newspaper and magazine)	27	3.6

^a^Multiple response

## Discussion

In this study, we investigated the prevalence of self-medication as well as the knowledge and perception towards self-medication practice among healthcare students in a Nigerian University. This was done to better understand the extent of this practice among the cohort, while exploring the reasons that drives or encourages such practice. Our findings reveal a moderately high prevalence (54.6%) of self-medication practice across the three major disciplines of healthcare students studied. The moderately high rate of self-medication practice is consistent with previous studies across different countries, which focus largely on university students and the general population [6–9, 11–15, 21, 23, 24, 26, 27, 33, 34]. The variation in self-medication practice among countries might be attributed to the differences in demographic characteristics and socioeconomic factors of respondents [6–9, 11–15, 21, 23, 24, 26, 27, 33, 34].

Of note, the self-medication practice was highest among female healthcare students compared to their male counterparts, which perhaps correlate with previous studies [10–13]. The gender difference in self-medication practice among the respondents might possibly be linked to the propensity of the females to engage in self-recommendation of analgesic group of medications to relieve pains and symptoms during their monthly menstrual cycle. Analgesics are the most commonly recommended medicines for the relief of menstrual pain in women, and as reflected in this study, menstrual pain is one of the top five conditions treated by self-medication.

In addition, 5th year Nursing and Pharmacy students, as well as Medicine and Surgery students constitute respondents who mostly engaged in self-medication practice. Generally, in the curriculum for the healthcare students at the 5th year, students are expected to have gained substantial knowledge in disease management, while they would have also been involved in direct patient care and management at one point or the other during their mandatory clinical rotations and exposure. Perhaps, this could have accounted for the perceived confidence in managing minor ailments by the respondents, as well as believe that they have acquired the requisite medical knowledge of what to use for a particular condition treated. Even though, ‘learning by doing’ is an encouraging concept that is consistently advocated in the training of healthcare students. However, for this approach to achieve the intended goal, it should be done responsibly, especially under the tutelage and guidance of senior and qualified clinical instructors/tutors in their respective disciplines. Otherwise, the act may lead to avoidable risk that self-medication could pose to future healthcare professionals. Previous studies have also documented that healthcare students tend to self-medicate probably owing to believe that they have the knowledge of what to use [6, 7, 12, 14].

In the present study, it is observed that pharmacy students engaged least in self-medication, compared to their counterparts in Nursing and Medicine, this variation in the engagement of self-medication could be as a result of pharmacy student knowing more about the risks of self-medication. Furthermore, female respondents, as well as nursing and medical students had higher odds of knowledge of self-medication practice than their other counterparts. Similarly, nursing and medical students had higher odds of perception of self-medication than the pharmacy students. Typically, one who have expected pharmacy students to have higher odds of knowledge and perception of self-medication than their nursing and medical students’ counterparts, mainly because they are largely going to be the future custodian of medicines, thus they should possess more knowledge of self-medication. This gap perhaps suggests a need to further look into the training curriculum of the healthcare students in general, and the pharmacy students in particular. This is in a bid to consider incorporation of relevant components of self-medication concept that will prepare the students for the future roles and responsibilities as a medicine expert.

Noteworthy to mention that the main source for purchasing medications for self-medication practices by respondents is the community pharmacies, followed by patent medicine vendors, while the left-over medicines at home, from friends and families were also mentioned. Hence, the need for appropriate government authorities specifically National Agency for Food Drug Administration and Control (NAFDAC) and the Pharmacists Council of Nigeria (PCN), to take necessary steps in ensuring consistent enforcement of medicine regulation as well as public enlightenment of responsible self-medication. On the other hand, community pharmacists especially should consistently uphold the ethics of the pharmacy profession by ensuring that only the clients who genuinely need a medicine obtain the product with proper guidance/instruction on rational usage. Our study is perhaps the first study in Nigeria to comprehensively evaluate self-medication practice among the healthcare students, while concurrently attempting to clarify the likely motivating reasons driving these practices, hence a major strength of the study.

Aside from analgesics as the most prevalent medication category used for self-medication, antimalarial and antibiotics were also part of the topmost medications used to self-medicate by respondents. This is not surprising, and can be supported by the fact that headache, malaria, cough, menstrual pain, cold and flu were the most frequently treated conditions by the respondents. Findings from previous studies also reported that analgesics and antipyretic are the most prevalent medication categories used for self-medication [7, 12, 15, 28, 35], Ideally, antibiotics and antimalarial should not be sold without a prescription from a qualified medical practitioner/physician as practiced in most developed countries and some developing countries. However, this is contrary to the situation in Nigeria, where most antimalarial and antibiotics may be purchased over-the-counter from medicine vendors without a prescription, and some community pharmacies are also inclusive. The weak enforcement of medicine regulations, as well as public easy accessibility to prescription-only medicine needs to be addressed by the appropriate authorities in order to prevent potential harm(s). Previous studies have reported that between 52.2% and 55% of Nigerian population obtained their medicines for self-medication practice from the patent medicine stores [23, 24], where they do not have the opportunity of accessing relevant and appropriate counselling services to guide medication usage [36, 37].

Our study also reveals that patient information leaflet is topmost of the common sources of information on dosage regimen for medicines used for self-medication practice. This may be expected since most of the respondents are more likely to be aware that patient information leaflet or the manufacturer’s literature insert serves as a quick and most readily available source of information for pharmaceutical products, though sometimes, manufacturer’s bias may distort/affect the reliability of information from such package insert.

### Limitation of the study

This study is limited by the possibility of recall bias, which is most inherent in self-report study of this nature. Also, the likelihood of respondents discussing among themselves while filling the questionnaire may not be totally excluded, being a self-administered questionnaire. In addition, future study may still need to consider other possible indicators aside from knowledge and perception as well as factors that may influence future healthcare professional especially pharmacy students in engaging in responsible self-medication practice. The above-mentioned gaps may therefore need to be considered in order to ensure a far-reaching conclusion, while caution should be exercised in generalizing the study findings to the entire population of healthcare students in the region.

## Conclusion

Prevalence of self-medication among the studied healthcare students is moderately high, while approximately half demonstrates good knowledge and perception of self-medication practices. Gender and respondents’ discipline were the notable independent predictors of good knowledge of self-medication, while stimulation for self-medication practice largely arise from the perception of treating minor ailments. This generally underscores a need for relevant advocacy and incorporation of aspects of responsible self-medication practice during the formal training of these future healthcare professionals. In addition, national guideline on medicine access should be re-enforced, with strong measures put in place to implement the policy, this may become necessary in order to avert the widespread negative consequences of self-medication practice among the populace generally.

## Data Availability

The datasets used and/or analyzed during the current study are available from the corresponding author on reasonable request.

## Abbreviations

NAFDAC: National Agency for Food and Drug Administration and Control
OTC: Over the counter
PCN: Pharmacist Council of Nigeria
WHO: World Health Organization
